# A 3-Year Study Reveals That Plant Growth Stage, Season and Field Site Affect Soil Fungal Communities while Cultivar and GM-Trait Have Minor Effects

**DOI:** 10.1371/journal.pone.0033819

**Published:** 2012-04-17

**Authors:** Silja Emilia Hannula, Wietse de Boer, Johannes van Veen

**Affiliations:** 1 Netherlands Institute of Ecology (NIOO-KNAW), Department of Microbial Ecology, Wageningen, The Netherlands; 2 Insititute of Biology, Leiden University, Leiden, The Netherlands; University of Missouri, United States of America

## Abstract

In this three year field study the impact of different potato (*Solanum tuberosum* L.) cultivars including a genetically modified (GM) amylopectin-accumulating potato line on rhizosphere fungal communities are investigated using molecular microbiological methods. The effects of growth stage of a plant, soil type and year on the rhizosphere fungi were included in this study. To compare the effects, one GM cultivar, the parental isoline, and four non-related cultivars were planted in the fields and analysed using T-RFLP on the basis of fungal phylum specific primers combined with multivariate statistical methods. Additionally, fungal biomass and some extracellular fungal enzymes (laccases, Mn-peroxidases and cellulases) were quantified in order to gain insight into the function of the fungal communities. Plant growth stage and year (and agricultural management) had the strongest effect on both diversity and function of the fungal communities while the GM-trait studied was the least explanatory factor. The impact of cultivar and soil type was intermediate. Occasional differences between cultivars, the amylopectin-accumulating potato line, and its parental variety were detected, but these differences were mostly transient in nature and detected either only in one soil, one growth stage or one year.

## Introduction

Genetic engineering of plants has been used to improve the quality and quantity of crop production in a cost-effective way (e.g. by enhancing resistance to pests and diseases or introducing tolerance to herbicides) [1]. Despite the great potential of this technology to advance agricultural yields, there are major concerns about the ecological impacts of genetically modified (GM) crops on soil ecosystem functioning. These impacts may be (1) direct (e.g. toxicity of an expressed introduced gene on key non-target species of important functional groups), (2) indirect (e.g. effects via unintended changes in the metabolism of the plant thereby affecting root exudates composition and fluxes) or (3) caused by changes in management regime used with GM crops [2].

The rhizosphere is a hot-spot of microbial abundance and metabolic activity due to the resources released by plants [3], [4]. Hence, possible side-effects of GM plants on functioning of soil microbes should be first considered for the rhizosphere. Together with bacteria, fungi in the rhizosphere are very important to functioning of the soil-plant system and their functions range from symbiotic arbuscular mycorrhizal fungi (AMF) and plant pathogens to decomposers [5], [6].

The structure and functioning of soil microbial communities is affected by soil type [7]–[9], plant growth stage [7], [10]–[13], and other abiotic and biotic factors such as agricultural management [14], [15]. The magnitude of the effects exerted by these factors compared to possible effects of cultivar and GM-crops is still largely unknown although knowledge of these sources of natural variation is critical for the assessment of the relative effects of specific potential perturbations such as introduced GM-traits.

Most of the studies on soil fungal communities have shown that GM-crops affect soil fungi in a similar way as its isoline [7], [13], [16]–[22], and only three studies [23]–[25] observed significant differences between the GM-variety and its parental isoline which could, however, be explained by factors other than GM-trait. Common to these studies was that the normal variability between cultivars under field conditions was usually very high and that other factors than cultivar-type affected the soil fungal communities more than the cultivar-type did. The aforementioned studies usually focused on one growth stage or one season/year without investigating variability over seasons. Thus, the question remains if different cultivars of potato, including a GM variety, have different effects on diversity or functioning of the soil microbes over multiple years.

Identifying the normal variation in fungal community structure and function in the soil is very important when aiming to evaluate the possible effects of GM-crops on soil communities [26]. In this study we followed the fungal community structure and function in two fields located in the Netherlands during 3 years of growing potatoes (*Solanum tuberosum* L.). Three growth stages of six cultivars (including a GM-variety with modified starch quality and its parental isoline) were included in the study allowing us to determine the long-term (years) and short term (within growth season) effects of the potato cultivars on fungal community dynamics and fungal decomposing activities. This approach facilitated an evaluation of the normal variation in fungal communities between years, growth stages, soils and under different cultivars, thereby providing a necessary baseline for assessing the potential impact of this GM potato variety. Further, we sampled the fields also after the growing seasons as well as in the rhizosphere of the succeeding crop (barley) to learn about possible long term effects of the starch-modified GM-potatoes.

## Materials and Methods

### Field Set-up and Sampling

Two agricultural sites VMD and BUI were selected for this experiment [19]. They are both located in the northern part of the Netherlands and are 10 km apart. Details on soil type, soil parameters and fertilizer treatments are presented in table S1. Cropping in these sites consists of potato-barley rotation (1 crop per year). Plots with six cultivars of potato were sampled in years 2008, 2009, and 2010 and barley fields were sampled after cultivation with potato in 2009. The fields were fertilized with 180–220 kg ha^−1^ nitrogen (N) in the form of calcium ammonium nitrate, 56–81 kg ha^−1^ phosphorous (P) as P_2_O_5_ and 145–200 kg ha^−1^ potassium (K) as K_2_O or K_2_SO_4_ in 2008 and 2009. In 2010 organic fertilizer in form of pig manure (14 ton ha^−1^in field VMD and 25 ton ha^−1^ in field BUI, respectively) was added together with inorganic fertilizers (table S1). Six cultivars of potato; ‘Aveka’, ‘Aventra’, ‘Désirée’, ‘Premiere’, ‘Karnico’ and ‘Modena’ (the modified variety of ‘Karnico’) were grown each in four replicates on these fields in randomized block design and locations were varied between years. The variety ‘Modena’ was genetically modified for its starch composition by complete inhibition of the production of amylose via introduction of a RNAi construct of the granule-bound starch synthase gene inhibiting GBSS and amylose formation, which yields pure amylopectin [27]. Cultivars ‘Aventra’, ‘Aveka’, ‘Karnico’ and ‘Modena’ produced tubers with a relatively high starch content and had a low to medium growth rate, whereas cultivars ‘Désirée’ and ‘Premiere’ had lower starch content in the tubers and higher growth rates.

Soil samples were collected from bulk soil before and after harvest whereas both rhizosphere and bulk soil were collected at the growth stages EC30 (seedling/young), EC60 (flowering) and EC90 (senescence) [28]. Bulk soil was collected using 0–15 cm soil corers (diameter 10 cm) and 5 cores per plot were randomly sampled and used to form a composite sample per plot that was further homogenized and sieved (4 mm mesh) to remove possible root fragments and stones. Rhizosphere soil was collected from a combination of 4 plants per plot by brushing roots. Part of the soil sample was subsequently frozen at −80°C for molecular analyses, another part was kept at −20°C prior to enzymatic analyses and ergosterol measurements and another part was used for immediate analyses of soil water content and pH (table S1). Soil water content was determined from fresh material as weight loss after overnight drying at 105°C.

### Enzymatic Analyses

Quantification of ergosterol, via the alkaline extraction method, was used as an estimate of fungal biomass [29]. Analyses of activities of enzymes involved in decomposition of lignocellulose-rich organic matter, *i.e*. laccase, cellulase and Mn-peroxidase were performed according to van der Wal *et al*. [30].

### Molecular Analyses

DNA was extracted from soil (0.5 g wet weight) with a Power Soil DNA isolation kit (MOBIO Laboratories, Inc. Carlsbad, CA, USA) using a bead beating system. Yields of genomic DNA were checked on 1% agarose gel and visualized under UV after ethidium bromide staining.

Terminal restriction fragment length polymorphism (T-RFLP) combined with the construction of a small library of the most dominant operational taxonomical units (OTUs) was used to determine the fungal community compositions over years. The structures of the three fungal phyla studied, ascomycetes, basidiomycetes and glomeromycetes, were assessed separately. For the analysis of ascomycete and basidiomycete communities, internal transcribed spacer (ITS) regions were used as target regions and the large subunit of ribosomal genes (LSU) was used as a target region for AMF (*Glomeromycota*). PCR conditions, primers and restriction enzymes are given in Hannula *et al.* [19]. Appropriate dilutions based on test runs of terminal restriction fragments (TRFs) were analyzed with an ABI 3130 sequencer using GeneScan™ −500 LIZ (Applied Biosystems) and used as a size standard.

Clone libraries were constructed as described in Hannula *et al.* [19] and partially the same clone libraries were used. The sequenced clones were assigned to OTUs based on comparisons with GenBank using BLAST and considered to belong to a genus or species with similarities of 95% for an order and 97% for a species. These OTUs were related to the orginal peaks and their presence and absence in field samples were evaluated in T-RFLP Analyses Matching Program (TRAMP-R) [31] in the statistical computing environment R. Three out of four of the enzyme/primer combinations within 1.5 bp margin had to be met in a sample for it to be assigned to an OTU.

### Data Analyses

Analyses of variance (ANOVA) with a linear mixed effect model was used to compare the ergosterol and enzymatic data as well as number of TRFs using SPSS for windows (Release 17.0.). The assumption of normality was tested with Shapiro-Wilk statistics and homogeneity of variances was assessed with Levene’s test. The field site, growth stage, year of sampling, cultivar and GM-variety were used as fixed factors and block was set as the random factor. Differences between treatments were compared by a post hoc Tukey’s honestly significant difference (HSD) test. Log transformation was used when data were not normally distributed. To estimate the possible effects of GM variety ‘Modena’ to its parental variety over years, a mixed model with repeated measure (growth stage) and block as a random factor was built separately for both fields.

The quality of T-RFLP data was first visually inspected in GeneMapper Software v4.1 (Applied Biosystems) and then transferred to T-Rex [32]. True peaks were identified for both labels as those of which the height exceeded the standard deviation (assuming zero mean) computed over all peaks and multiplied by two [33]. Non-Metric Multidimensional Scaling (NMDS) with Jaccard as distance measure were used to assess the similarity of the fungal communities after the harvest and in the rhizosphere of next crop, barley. Principal component analyses (PCA) were used to analyse the communities between years, fields, growth stages and cultivar. The community fingerprints were compared using ANOSIM in PAST [34]. In short, ANOSIM is a non-parametric test of significant differences between groups by comparing distances between groups to distances within groups. We used Jaccard as a distance index and 10000 permutations. Pairwise ANOSIMs between field sites, growth stages, years and cultivars are provided.

The diversity was calculated from the matched samples with both Shannon-H’ and Simpson diversity indexes and compared with ANOVA as explained above.

## Results

### Soil Enzymatic Analyses, Fungal Biomass and Fungal Richness

**Figure 1:**
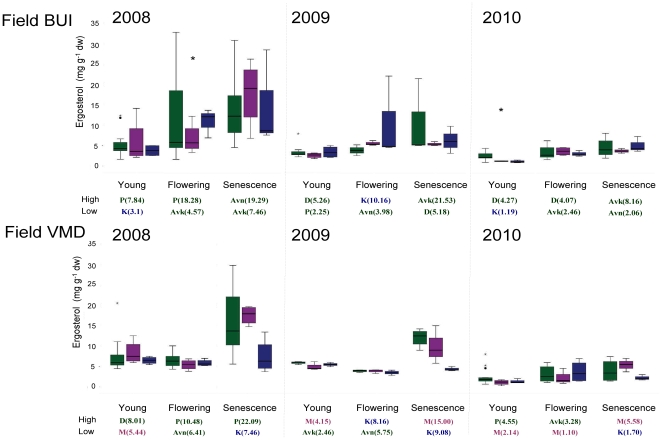
Change in fungal biomass. Boxplots of fungal biomass in the rhizosphere as measured by ergosterol concentrations during 3 years in different growth stages and in both field locations. The baseline (all other cultivars combined, n = 16) is marked with green boxplots, the GM-variety (n = 4) with purple and the parental variety ‘Karnico’ (n = 4) with blue markers. The star indicates a significant cultivar effect at the indicated time point. The values under the graphs are the cultivars with highest and lowest values (on average) colored the same as in the boxplots where ‘D’ = ‘Désirée’, ‘Avk’ = ’Aveka’, ‘Avn’ = ’Aventra’, ‘P’ = ‘Premiere’, ‘K’ = ‘Karnico’ (parental cultivar) and ‘M’ = ’Modena’ (modified cultivar).

Fungal-related parameters in plots cropped with the GM-variety seemed to fall within normal variation among potato cultivars observed in time (table 1). The largest explaining factor for most of the measured parameters was the plant phenological growth stage, followed by year and the soil type (table 1). Ergosterol analyses indicated that soil fungal biomass strongly dependent on plant growth stage and varied from year to year (table 1, Fig. 1). Although growth stage was affecting the fungal biomass, there were no significant differences between pre- and post-cropping situations or in bulk soils (F = 1.31, p = 0.25). Hence, no long term effects of cultivation were detected. Cultivar did not affect the fungal biomass in the rhizosphere in general, however, differences between some cultivars were detected in pairwise comparisons: cultivar ‘Premiere’ had a significantly lower fungal biomass as assayed by the ergosterol method in its rhizosphere than cultivars ‘Aveka’ and ‘Désirée’ (F = 4.131 and 4.181, p<0.05) over the entire period. In field BUI significant effects of cultivar on fungal biomass were detected at the stage of flowering in 2008 and the stage of young plant in 2010 (table 2) while in field VMD there were no effects of cultivar at any stage. Furthermore, there was no consistency in cultivars having the lowest or highest amount of ergosterol in their rhizosphere (Fig.1). The GM cultivar ‘Modena’ was not significantly different from the other cultivars or the parental variety (table 2) but rather in the middle range of the cultivars in the field BUI. The only significant difference between the GM-variety and its parental variety was the amount of ergosterol in the rhizosphere in the senescent stage (table 2).

**Table 1:** ANOVA comparisons of several fungal-related parameters between fields, years, growth stages, cultivars and GM-trait and the interaction effects of the cultivar.

	Field (df. 1)		Year (df.2)		Growth stage (df. 3)		Cultivar (df. 5)		GM- parent (df. 1)		Year×cultivar		Field×cultivar		Growth stage×cultivar		Year×Growth stage×field×cultivar	
	*F*	*P*	*F*	*P*	*F*	*P*	*F*	*P*	*F*	*P*	*F*	*P*	*F*	*P*	*F*	*P*	*F*	*P*
Ergosterol (mg/g)	0.13	0.72	48.17	**<0.001**	19.38	**<0.001**	1.47	0.20	0.12	0.73	1.40	0.18	1.00	0.42	0.97	0.49	1.72	0.071
Laccases (µmol/g)	0.63	0.43	14.39	**<0.001**	21.19	**<0.001**	1.05	0.39	0.36	0.55	1.84	0.052	1.27	0.28	2.39	**0.004**	1.72	0.052
Mn-Peroxidases (µmol/g)	1.06	0.10	1.96	0.14	9.81	**<0.001**	3.31	0.06	0.67	0.42	1.02	0.43	1.07	0.38	1.86	**0.031**	1.69	**0.043**
Cellulases (µmol/g)	17.74	**<0.001**	23.94	**<0.001**	19.01	**<0.001**	1.08	0.37	0.04	0.83	4.03	**<0.001**	3.96	**0.002**	3.12	**<0.001**	1.29	0.35
# of Ascomycetes	0.41	0.52	6.28	**<0.001**	25.15	**<0.001**	1.51	0.19	2.73	0.11	0.72	0.69	0.48	0.79	2.67	**0.001**	1.38	0.16
# of Basidiomycetes	1.65	0.20	51.60	**<0.001**	20.14	**<0.001**	0.72	0.61	0.16	0.69	0.52	0.88	0.08	1.00	1.12	0.34	0.39	0.97
# of AMF	0.61	0.44	15.29	**<0.001**	6.09	**<0.001**	0.66	0.65	0.35	0.55	0.49	0.88	0.89	0.49	0.34	0.98	0.50	0.91

Significant P-values are marked with bold. Only samples from rhizosphere were included in analyses of growth stage, cultivar and GM-parent comparison. # indicates richness of the fungi.

**Table 2:** ANOVA analysis of effects of cultivar (including all cultivars) and GM-cultivar ‘Modena’ versus parental cultivar ‘Karnico’on fungal biomass, enzymatic activities and fungal richness in the rhizosphere in different fields, years and growth stages.

				Ergosterol (mg/g)		Laccases (µmol/g)		Mn-Peroxidases (µmol/g)		Cellulases (µmol/g)		# of Ascomycetes		# of Basidiomycetes		# of AMF	
				Cultivar	GM-Parent	Cultivar	GM-Parent	Cultivar	GM-Parent	Cultivar	GM-Parent	Cultivar	GM-Parent	Cultivar	GM-Parent	Cultivar	GM-Parent
			**df.**	**5**	**1**	**5**	**1**	**5**	**1**	**5**	**1**	**5**	**1**	**5**	**1**	**5**	**1**
**Field BUI**	**2008**	Young	*F*	0.86	0.24	0.89	2.99	1.02	1.32	0.48	0.92	0.22	0.06	0.90	0.05	1.09	0.10
			*P*	0.53	0.65	0.51	0.15	0.44	0.30	0.79	0.38	0.95	0.82	0.50	0.83	0.42	0.78
		Flowering	*F*	**12.64**	2.13	1.00	0.59	0.76	0.42	0.79	0.00	0.38	0.25	0.48	0.02	1.89	0.47
			*P*	**<0.001**	0.20	0.44	0.47	0.59	0.84	0.57	0.96	0.86	0.63	0.79	0.89	0.15	0.53
		Senescence	*F*	2.07	0.15	2.61	0.20	**9.19**	0.85	1.25	0.51	**3.67**	**39.06**	0.66	0.38	1.93	0.28
			*P*	0.12	0.72	0.06	0.67	**<0.001**	0.40	0.33	0.50	**0.018**	**0.025**	0.66	0.57	0.15	0.62
	**2009**	Young	*F*	1.22	1.13	1.24	0.50	0.73	0.61	0.94	0.61	0.13	0.07	0.47	0.19	1.12	2.42
			*P*	0.36	0.33	0.35	0.51	0.59	0.46	0.47	0.46	0.97	0.80	0.75	0.69	0.40	0.17
		Flowering	*F*	1.04	0.72	0.45	2.63	1.00	0.34	0.69	5.53	0.17	3.00	0.87	0.00	0.04	5.44
			*P*	0.41	0.45	0.77	0.16	0.45	0.58	0.62	0.06	0.85	0.33	0.49	0.96	0.96	0.26
		Senescence	*F*	1.32	0.20	1.68	1.32	1.42	1.36	1.80	0.71	0.32	1.93	1.60	2.10	1.24	1.15
			*P*	0.32	0.68	0.26	0.33	0.33	0.33	0.23	0.46	0.81	0.21	0.27	0.24	0.37	0.40
	**2010**	Young	*F*	**9.49**	0.01	1.09	0.11	0.11	0.26	1.32	0.49	0.92	1.94	0.84	1.26	0.34	0.35
			*P*	**0.001**	0.94	0.41	0.75	0.99	0.63	0.31	0.52	0.49	0.21	0.54	0.31	0.88	0.58
		Flowering	*F*	0.85	0.77	1.06	0.64	0.84	0.69	0.47	0.13	0.19	0.02	2.52	0.06	0.34	1.08
			*P*	0.54	0.42	0.41	0.46	0.54	0.80	0.79	0.73	0.96	0.89	0.31	0.81	0.88	0.49
		Senescence	*F*	4.73	1.42	0.65	0.22	1.89	0.30	1.55	1.51	0.33	1.04	0.68	0.00	1.53	**6.42**
			*P*	0.11	0.30	0.63	0.66	0.17	0.61	0.24	0.27	0.86	0.38	0.62	0.95	0.24	**0.044**
**Field VMD**	**2008**	Young	*F*	1.96	1.49	1.20	1.21	0.76	0.09	1.03	0.34	2.13	2.50	0.34	0.10	0.55	1.54
			*P*	0.13	0.27	0.35	0.31	0.59	0.78	0.43	0.58	0.11	0.17	0.89	0.77	0.74	0.26
		Flowering	*F*	0.67	0.33	1.43	1.19	0.73	0.65	1.23	1.93	0.56	0.33	0.46	1.47	2.10	1.03
			*P*	0.65	0.59	0.27	0.32	0.61	0.46	0.35	0.21	0.73	0.59	0.72	0.44	0.12	0.35
		Senescence	*F*	1.43	**17.50**	1.24	0.51	2.43	0.18	0.81	0.10	0.66	1.93	0.56	0.26	0.66	0.93
			*P*	0.26	**0.006**	0.33	0.50	0.08	0.69	0.56	0.76	0.66	0.21	0.73	0.65	0.66	0.38
	**2009**	Young	*F*	2.91	0.54	0.66	0.22	0.27	0.06	0.32	0.15	0.57	0.24	0.58	0.36	1.06	0.62
			*P*	0.17	0.50	0.59	0.66	0.85	0.82	0.81	0.72	0.65	0.64	0.64	0.57	0.40	0.46
		Flowering	*F*	7.03	0.62	0.98	0.18	**4.24**	1.18	1.22	2.83	0.67	2.00	0.25	0.85	1.58	0.29
			*P*	0.23	0.47	0.44	0.69	**0.03**	0.32	0.35	0.14	0.54	0.22	0.90	0.41	0.29	0.63
		Senescence	*F*	0.77	**14.16**	1.57	1.18	1.35	1.24	0.36	0.01	1.35	1.04	**6.00**	4.09	2.38	5.89
			*P*	0.68	**0.011**	0.23	0.32	0.30	0.31	0.84	0.92	0.36	0.38	**0.03**	0.11	0.13	0.06
	**2010**	Young	*F*	0.86	0.30	0.83	1.05	0.65	2.97	0.68	0.93	0.53	0.18	0.83	1.67	**4.01**	4.17
			*P*	0.53	0.61	0.55	0.34	0.66	0.14	0.65	0.37	0.75	0.68	0.55	0.24	**0.03**	0.11
		Flowering	*F*	0.75	0.99	1.76	1.00	2.13	1.00	0.78	0.86	1.08	0.74	0.25	0.09	2.02	8.17
			*P*	0.60	0.36	0.17	0.36	0.11	3.56	0.58	0.39	0.41	0.42	0.90	0.78	0.18	0.07
		Senescence	*F*	0.27	**13.59**	1.54	1.17	0.18	0.13	0.30	0.06	0.95	0.46	0.47	1.02	0.12	0.52
			*P*	0.84	**0.014**	0.30	0.54	0.90	0.74	0.82	0.82	0.45	0.53	0.71	0.35	0.95	0.51

Significant P-values are marked with bold.

Correlations revealed that all the extracellular enzymes measured in this study (laccases, cellulases and Mn-peroxidases) were positively correlated with the fungal biomass indicator ergosterol (n = 702, R^2^ between 0.23–0.29 and p<0.001). Further, there were strong positive correlations among all enzyme activities measured. The richness of both ascomycetes and basidiomycetes was positively correlated with the amount of ergosterol (for basidiomycetes R^2^  =  0.27 and P<0.001 and ascomycetes R^2^ = 0.08 and P<0.05). AMF richness was negatively correlated with the amount of ergosterol (R^2^ = .11 and P<0.05). Furthermore, the amount of Mn-Peroxidases in the soil was positively correlated with the ascomycete diversity (R^2^ = 0.16, P<0.001) while the AMF richness was negatively correlated with production of cellulases (R^2^ = 0.11 and P<0.005).

The measured extracellular enzymes (laccases, Mn-peroxidases and cellulases) were all affected by plant growth stage; highest activities were measured during senescence (table 1, Fig. 1). The amount of laccases and cellulases in the rhizosphere was significantly affected by year and the highest activity of these enzymes was found in 2009. On average the BUI location had higher laccase and cellulase activity than field VMD. The amount of Mn-peroxidases was associated with cultivar, but other enzymes were not. The cultivar ‘Modena’ had similar amounts of Mn-peroxidase in its rhizosphere as the parental cultivar ‘Karnico’, but more Mn-peroxidases in its rhizosphere than was found in the rhizospheres of Premiere and Aveka.

When looking at individual time points and fields the ascomycete, basidiomycete and glomeromycete richness was only once significantly different between cultivars (table 2). The richness of ascomycetes and glomeromycetes in the rhizosphere of GM-cultivar was only once different from the parental cultivar, namely at senescence 2008 and senescence 2010 in field BUI. The basidiomycete richness was at no occassion different between GM- and parental cultivar (table 2).

**Figure 2:**
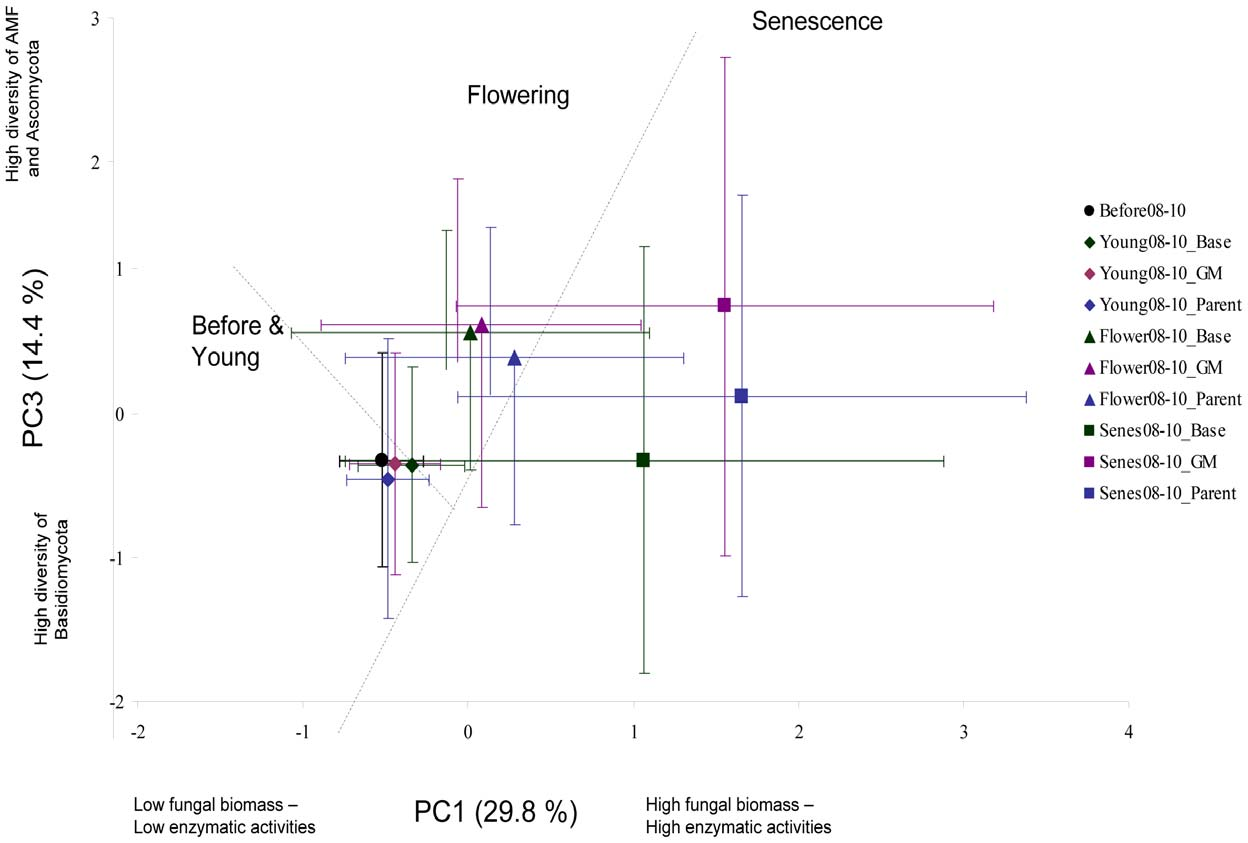
Principal component analysis of functioning and diversity of fungal communities in plots cropped with different potato cultivars. For clarity, the years and field sites are combined. Pre-cropping samples are represented by black circles, young plant stage samples with diamonds, flowering plants stage samples with triangles and senescence stage samples with squares. Green markers and error bars represent baseline cultivars (n = 96), purple markers the GM-variety (n = 24) and blue markers the parental variety ‘Karnico’ (n = 24). The explanatory parameters are mentioned next to the axis. The enzymes measured as functional parameters were laccases, Mn-peroxidases and cellulases.

Data on community function, as based on activities of enzymes involved in decomposition of lignocellulose-rich organic matter, and richness were analysed by principal component analyses (PCA). The PCA analyses revealed that the growth stage was the strongest explanatory factor of differences in the community function (Fig. 2). The stage senescence clearly separated from the other stages along PC1 (ANOVA; F  =  9.57–13.74, p<0.001) which was explained with higher ergosterol and enzymatic activities during senescence. The PC2 was explained by the same factors as PC1 and is thus not used here. The flowering stage separated along PC3 (F = 4.22–8.28, p<0.05) which is explained by more AMF and ascomycetes and less basidiomycetes during that stage compared to the other stages. Further, the years separated along both axes (PC1: F = 8.5, p<0.001 and PC3: F = 124.6, p<0.001) and fields along PC3 (F = 33.9, p<0.001) (Fig. S1). Cultivar had no significant contribution to explanation of PC1 (F = 1.83, P = 0.15), PC2 (F = 1.92, P = 0.12) nor PC3 (F = 0.88, P = 0.47) and the GM-variety was not significantly different from its parental isoline ‘Karnico’ (Fig. 2).

### Fungal Diversity and Community Structure

**Figure 3:**
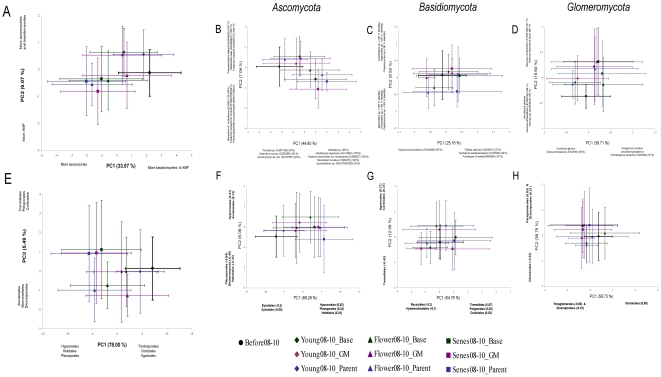
Principal component analysis of community structure of identified fungi. The PCA analysis was done both at the level of individual OTUs and of orders for total fungi (A & E), *Ascomycota* (B & F), *Basidiomycota* (C & G) and *Glomeromycota* (D & H). Figures A–D depict the identified fungal OTUs whereas figures E–H indicate the levels of orders. Orders together with identified OTUs are given in table S1. For clarity, the years and field sites are combined. Pre-cropping soil samples are marked with black circles, young plants stage with diamonds, flowering plant stage with triangles and senescence stage with squares. Green markers and error bars represent baseline cultivars (n = 96), purple markers the GM-variety (n = 24) and blue markers the parental variety ‘Karnico’ (n = 24). The OTUs (figures A–D) and orders (E–H) that do significantly explain the components are mentioned next to the axis.

According to the ANOSIM, the community fingerprints of all TRF peaks as well as identified OTUs of *Ascomycota*, *Basidiomycota* and *Glomeromycota,* were affected by the growth stage of the plant, field site and year (Fig. 3, Table 3). The fungal community structure was most strongly influenced by year-to-year variation (R>0.22) and difference in growth stage (R>0.09). The R values for the field site were close to 0 however, due to the size of the data-set a significant difference between fields were found. Plant cultivar did not predict fungal community structure when all growth stages, years and both fields were considered together (Table 3). There were no significant differences in the community structure of ascomycetes, basidiomycetes, glomeromycetes or total fungi between GM-cultivar ‘Modena’ and its parental variety ‘Karnico’ in any pairwise comparisons (Fig. 3).

**Table 3:** ANOSIM comparisons between the fields, years, growth stages, cultivars and GM-trait for *Ascomycota*, *Basidiomycota* and *Glomeromycota*.

	Field		Year		Growth stage		Cultivar*		GM-parent*	
	R	P	R	P	R	P	R	P	R	P
*Ascomycota*	0.07	**<0.001**	0.29	**<0.001**	0.10	**<0.001**	0.013	0.131	−0.006	1
*Basidiomycota*	0.04	**<0.001**	0.25	**<0.001**	0.19	**<0.001**	0.008	0.188	0.015	0.915
*Glomeromycota*	0.11	**<0.001**	0.22	**<0.001**	0.09	**<0.001**	−0.005	0.689	−0.011	0.863

* Only samples where plant was present are included in the analyses.

Significant P-values are marked with bold.

The diversity of all fungal phyla was expressed both by the Shannon-Wiener index (H’) and Simpson diversity index. The ascomycete diversity was significantly correlated with ascomycete richness (R^2^ = 0.55 for total diversity, R^2^ = 0.45 for orders and R^2^ = 0.36 for classes, P<0.001 for all) and basidiomycete diversity with basidiomycete richness (R^2^ = 0.51 for total diversity and R^2^ = 0.41 for orders, P<0.001 for both). Further, the ascomycete diversity was negatively correlated with basidiomycete diversity (R^2^ = −0.15, P<0.005). Ascomycete richness was correlated with the amount of Mn-peroxidases in the soil (R^2^ = 0.15, P<0.05) and basidiomycete richness with ergosterol (R^2^ = 0.18, P<0.001). The AMF diversity was positively correlated with soil moisture content (R^2^ = 0.15, P<0.001), AMF richness (R^2^ = 0.58, P<0.001) and ascomycete diversity (R^2^ = 0.10, P<0.05).

**Figure 4:**
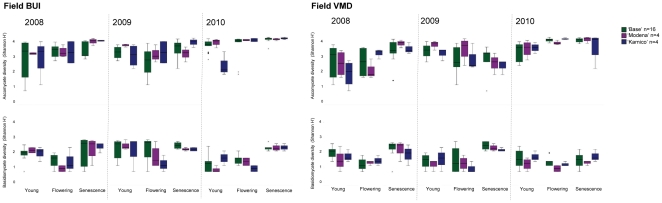
Effect of cultivar, year, growth stage and field on fungal diversity. Boxplots of changes in diversity of *Ascomycota* and *Basidiomycota* between years, growth stages, fields and between baseline, GM and its parental variety. The baseline (all other cultivars combined, n = 16) is marked with green boxplots, the GM-variety (n = 4) with purple and the parental variety ‘Karnico’ (n = 4) with blue markers. Diversity was calculated using Shannon-Wiener index (H’) and statistical comparisons are presented in table 5.

The diversity of ascomycetes or basidiomycetes at the level of OTUs or orders was not significantly affected by field site. However, AMF diversity was. There was no significant difference in diversity of ascomycetes at the level of OTUs and orders from year to year, although diversity between years 2009 and 2010 was significantly different. However, at the level of classes also 2008 and 2009 were different and year was a more pronounced factor explaining the diversity. For basidiomycetes and AMF, year had a strong influence on diversity both at the level of OTUs and orders (table 4). Growth stage, had a strong significant effect on ascomycete and basidiomycete diversities (Fig. 4, table 4) but less effect on the AMF diversity in the rhizosphere.

**Table 4:** The effect of field site, year, growth stage and cultivar on soil ascomycete, basidiomycete and glomeromycete diversity for different taxonomic levels.

		Field		Year		Growth stage		Cultivar*		GM-parent*	
		F	P	F	P	F	P	F	P	F	P
*Ascomycota*	OTUs	0.005/0.004	0.94/0.94	7.80/3.89	**<0.001/0.02**	12.76/9.16	**<0.001**	0.65/0.32	0.66/0.91	2.67/0.49	0.11/0.49
	Orders	0.33/0.009	0.57/0.92	7.44/3.56	**<0.005/0.03**	10.8/13.22	**<0.001**	0.59/0.52	0.64/0.76	2.74/1.58	0.10/0.21
	Classes	9.30/9.50	**0.03/0.02**	10.80/9.64	**<0.001**	6.78/5.76	**<0.001**	15.58/34.61	**<0.001**	2.97/2.31	0.09/0.31
*Basidiomycota*	OTUs	1.803/0.523	0.18/0.47	9.49/6.64	**<0.001/0.002**	13.84/9.37	**<0.001**	1.24/1.41	0.29/0.23	0.03/0.02	0.87/0.90
	Orders	0.04/0.002	0.85/0.97	21.85/17.86	**<0.001**	8.99/6.48	**<0.001**	1.85/2.08	0.13/0.09	0.19/0.37	0.67/0.54
*Glomeromycota*	OTUs	14.67/15.04	**<0.001**	24.48/20.72	**<0.001**	3.01/2.76	**0.03/0.04**	1.91/1.63	0.09/0.15	1.91/1.40	0.17/0.24
	Orders	38.22/35.98	**<0.001**	12.50/9.99	**<0.001**	2.29/2.13	0.08/0.09	1.91/1.89	0.09/0.10	1.17/1.59	0.19/0.21

* Only samples where plant was present are included in the analyses.

All diversities were calculated using both Shannon H’ and Simpson diversity indexes and presented in the table as Shannon H’/Simpson diversity. If both P-values are the same, only one value is presented. Diversity index for classes was not calculated for basidiomycetes and glomeromycetes due to low numbers or unevenness of classes. Significant P-values are marked with bold.

Cultivar-type had no overall effect on basidiomycete, ascomycete and AMF diversity at the level of OTUs or orders. However, at the level of classes cultivar ‘Désirée’ had a significantly less diverse community of ascomycetes in its rhizosphere than all the other cultivars causing a general cultivar effect (table 4). When the field sites, growth stages and years were considered separately, cultivar was a weak explanatory factor for the diversity of ascomycetes, basidiomycetes and AMF (Fig. 4, table 5). Both cultivar and GM-variety had an effect on diversity of ascomycetes in the rhizosphere in field BUI 2010 in the young-plant stage where ‘Karnico’ had a low diversity. The GM-variety had a significantly less diverse community of ascomycetes compared to its parental variety in field VMD 2010 at the stage of flowering plants (table 5). Basidiomycete diversity was different in rhizospheres between cultivars both during flowering and senescence 2009 in field VMD but never between GM and its parental cultivar. For AMF effects of cultivar and GM-variety were observed only at the first sampling moment of rhizosphere field in VMD (young 2008).

**Table 5:** ANOVAs of the effect of cultivar (including all cultivars) and GM-cultivar ‘Modena’ versus parental cultivar ‘Karnico’ on diversity of ascomycetes, basidiomycetes and glomeromycetes in the rhizosphere in both fields, all years and growth stages.

				Diversity of Ascomycota (Shannon H)			Diversity of Basidiomycota (Shannon H)			Diversity of Glomeromycota (Shannon H)		
				Cultivar	GM-Parent	Order level	Cultivar	GM-Parent	Order level	Cultivar	GM-Parent	Order level
				df. 5	df. 1		df. 5	df. 1		df. 5	df. 1	
**Field BUI**	**2008**	Young	*F*	0.12	0.12		0.21	0.60		0.38	0.38	
			*P*	0.89	0.75	n.s.	0.95	0.48	n.s.	0.86	0.85	n.s.
		Flowering	*F*	0.92	0.00		0.79	0.56		0.62	2.34	
			*P*	0.91	1.00	n.s.	0.58	0.50	n.s.	0.69	0.22	n.s.
		Senescence	*F*	2.93	0.05		0.99	0.30		0.93	0.60	
			*P*	0.11	0.83	n.s.	0.47	0.61	n.s.	0.50	0.50	n.s.
	**2009**	Young	*F*	0.88	1.22		0.23	0.40		0.99	0.88	
			*P*	0.51	0.52	n.s.	0.92	0.55	n.s.	0.46	0.38	n.s.
		Flowering	*F*	2.86	1.14		3.28	0.21		0.64	0.71	
			*P*	0.14	0.35	n.s.	0.10	0.63	n.s.	0.56	0.44	n.s.
		Senescence	*F*	1.35	3.29		4.88	1.19		nd	nd	
			*P*	0.35	0.21	n.s.	0.77	0.29	n.s.	nd	nd	n.s.
	**2010**	Young	*F*	**6.25**	**13.80**	**3.83/11.27**	0.79	5.98		0.54	0.60	
			*P*	**0.03**	**0.01**	**0.02/0.001**	0.58	0.07	n.s.	0.74	0.50	n.s.
		Flowering	*F*	0.46	0.00		2.86	2.24		0.43	0.13	
			*P*	0.80	0.98	n.s.	0.14	0.38	n.s.	0.82	0.74	n.s.
		Senescence	*F*	1.37	1.14		0.31	0.29		4.41	0.82	
			*P*	0.30	0.35	n.s.	0.86	0.82	n.s.	0.99	0.40	n.s.
**Field VMD**	**2008**	Young	*F*	1.73	1.89		0.45	0.19		**4.77**	**38.37**	**2.38/40.38**
			*P*	0.19	0.49	n.s.	0.80	0.69	n.s.	**0.01**	**0.00**	**0.02/0.001**
		Flowering	*F*	0.85	4.35		0.43	0.37		2.23	0.41	
			*P*	0.52	0.17	n.s.	0.79	0.58	n.s.	0.15	0.59	n.s.
		Senescence	*F*	0.99	0.04		0.57	0.34		0.13	0.32	
			*P*	0.48	0.86	n.s.	0.73	0.59	n.s.	0.93	0.62	n.s.
	**2009**	Young	*F*	0.64	1.79		0.45	0.26		0.51	0.20	
			*P*	0.62	0.41	n.s.	0.73	0.64	n.s.	0.69	0.67	n.s.
		Flowering	*F*	0.64	1.08		**4.95**	0.90		0.47	0.71	
			*P*	0.61	0.41	n.s.	**0.04**	0.39	n.s.	0.76	0.44	n.s.
		Senescence	*F*	0.35	1.21		**5.54**	0.93	**4.37/**1.61	2.31	23.78	
			*P*	0.84	0.44	n.s.	**0.02**	0.38	**0.04/**0.26	0.15	0.16	n.s.
	**2010**	Young	*F*	0.49	0.42		0.62	2.92		1.12	1.89	
			*P*	0.78	0.84	n.s.	0.66	0.15	n.s.	0.56	0.49	n.s.
		Flowering	*F*	2.66	**7.84**	1.43**/1.17**	2.72	2.34		0.46	0.33	
			*P*	0.08	**0.05**	0.28**/0.03**	0.13	0.22	n.s.	0.77	0.67	n.s.
		Senescence	*F*	0.84	0.91		0.54	2.87		0.49	0.42	
			*P*	0.50	0.38	n.s.	0.67	0.17	n.s.	0.75	0.86	n.s.

The diversities were estimated using Shannon-H’. The first two columns of each fungal group are performed at the level of OTUs and the third column indicates significance at the level of orders. Significant P-values are marked with bold.

### Legacy of GM-crops

**Figure 5:**
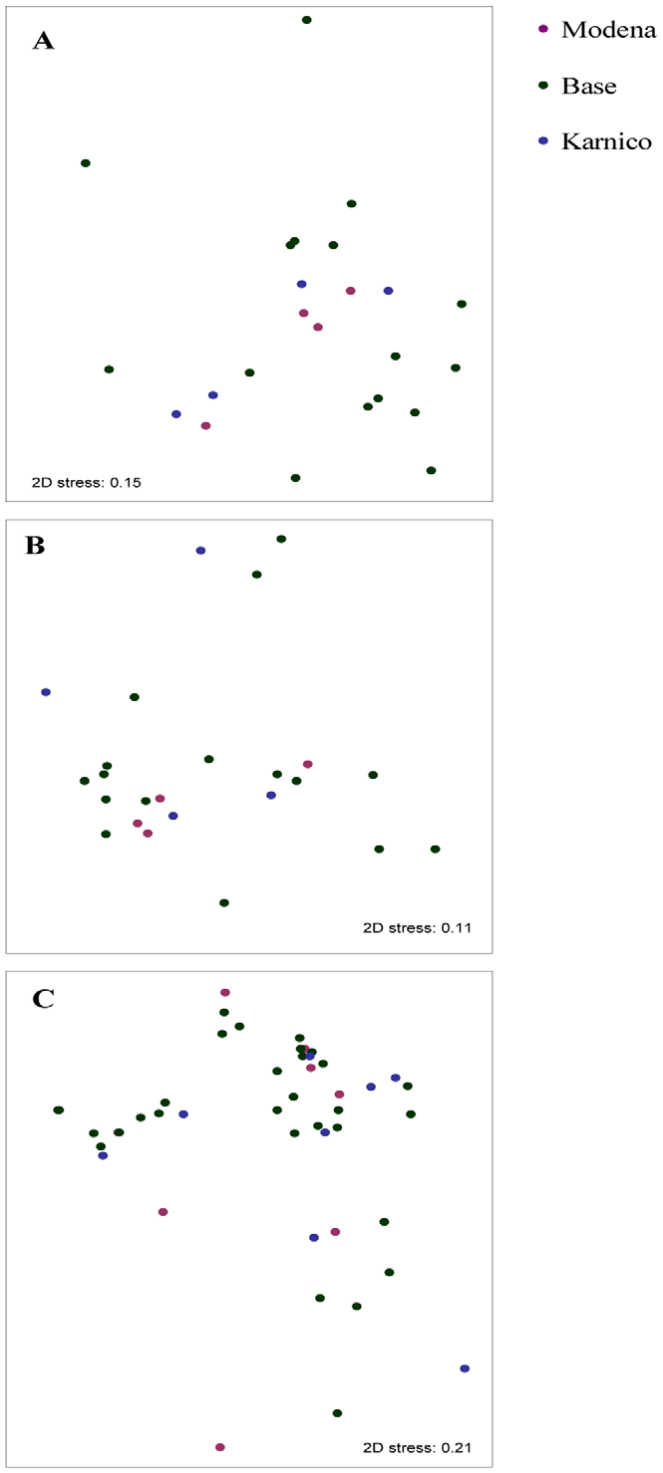
Long term effect of GM-trait on fungal community. NMDS of effects of GM-variety in the next crop (barley) rhizosphere in field BUI on ascomycetes (A), basidiomycetes (B) and in fields BUI and VMD on glomeromycetes (C). The GM-variety ‘Modena’ is marked with purple markers, the parental cultivar ‘Karnico’ with blue markers, and baseline (all other cultivars combined) green markers. Details on statistical analysis are given in table 6.

The fields were sampled after the growth seasons 2008 and 2009 and, in addition, rhizosphere of barley was sampled in June 2009 in the field where potatoes were grown in 2008. There were no significant differences in ergosterol content, enzymatic activities, fungal richness or fungal diversity between soils where ‘Modena’ and ‘Karnico’ had been grown (table 6). In the rhizosphere of barley there was no effect of previous genotype detected at all. Furthermore, no effect could be detected of different potato genotypes on the fungal community fingerprints in post-harvest samples and in the rhizosphere of barley (Fig. 5, table 6).

**Table 6:** Statistical analysis of the effects of cultivar and GM-trait on fungal-related parameters in post-harvest soil samples as well as in the rhizosphere of next plant barley.

		Field BUI				Field VMD				Barley rhizosphere			
		Cultivar		GM-parent		Cultivar		GM-parent		Cultivar		GM-parent	
		F/R	P	F/R	P	F/R	P	F/R	P	F/R	P	F/R	P
ANOVA	Ergosterol	0.14	0.89	0.00	0.95	0.81	0.47	1.04	0.35	0.03	0.98	0.00	0.98
	Laccases	0.70	0.63	0.28	0.62	3.63	**0.02**	0.06	0.82	0.56	0.58	1.03	0.36
	Mn-Peroxidases	1.77	0.19	2.76	0.15	1.30	0.31	0.00	0.98	0.00	0.99	0.00	0.99
	Cellulases	1.00	0.43	0.35	0.58	1.56	0.22	5.53	0.06	0.06	0.95	0.05	0.84
	Diversity of basidiomycetes									1.37	0.29	0.06	0.82
	Diversityof ascomycetes	0.87	0.72	0.33	0.64	0.34	0.72	0.58	0.48	0.72	0.51	0.60	0.48
	Diversity of AMF	0.61	0.50	0.02	0.89	0.53	0.68	1.00	0.36	0.55	0.60	1.14	0.35
ANOSIM	Community of ascomycetes	−0.22	0.88	−0.32	1.00	−0.05	0.72	0.07	0.24	0.01	0.42	0.56	0.33
	Community of basidiomycetes	0.03	0.65	−0.12	0.95	−0.13	0.73	−0.38	0.97	−0.24	0.91	−0.58	1.00
	Community of AMF	−0.04	0.67	−0.16	0.80	0.02	0.37	−0.13	0.81	−0.28	1.00	−0.38	1.00

ANOVA was used as a similarity measure for fungal biomass, enzymatic measurements and diversity and F values are presented in the table. ANOSIM was used for the community data derived from T-REX and R-values are presented in the table. Significant P-values for both ANOVA and ANOSIM are marked with bold.

## Discussion

The composition and function of fungal communities in the rhizosphere was shown to be highly dynamic and influenced by plant growth stage, soil type, year and, to a smaller extent, also cultivar-type. The largest explaining factor for most of the measured parameters was plant phenological growth stage, followed by year and the soil type. In addition, results confirmed our previous observations that fungal composition and abundance is strongly influenced by the presence of potato roots (i.e. a strong rhizosphere effect) [19].

The succession of microbial communities during plant growing season can be explained by two possible mechanisms [7]. The first one is related to temporal changes in abiotic conditions such as soil moisture and temperature. However this is not a likely option to explain the fungal community dynamics observed in this study as the three years of study were very contrasting in temperature and moisture. The second, more likely, mechanism is the changes in quality and quantity of root exudates and rhizodeposits with growth stage [35], [36] and or changes in root morphology. Although root exudates were not measured in this study, there is evidence of the effect of plant growth stage on root exudate fluxes which in turn affect soil microbial communities [8], [37]. Earlier studies indicated that bacterial and fungal communities in the rhizosphere would either decrease [7], [13], [20], [38] or increase [10], [11], [39]–[41] during plant maturation. Our results clearly indicate that the plants at the senescence stage (EC90) harbor the most diverse, active and abundant fungal communities. The presence of the highest fungal biomass and diversity at the stage of senescence was expected, as decomposable material (dead roots and leaves) is already available while root exudation still continues thereby broadening the spectrum of substrate availability [42]. Yet, the increase and magnitude of the fungal biomass and its activity in the rhizosphere at that stage is surprising. Until now, the prevailing belief was that the fungal biomass is low in soils under intensive agricultural management. Earlier results with the same cultivars under controlled conditions confirm our observations [43].

Surprisingly, despite the strong differences in soil organic matter content, field location did not affect the community function or diversity of the higher fungi much and results from the two fields could be even combined for baseline purposes. Earlier studies have found soil type as one of the most explanatory factor [7]–[9], [12], [18] affecting soil microbial communities. Bacterial communities appeared to differ strongly between the two fields used in this study, both for bulk soil and rhizosphere [44]. In our study, however, only total fungal community structure and diversity of AMF were strongly affected by the field site while fungal biomass and functional parameters such as enzymatic activities seemed to respond to the field type only slightly. The difference in AMF between fields could be probably explained by the higher organic matter content and thus higher AMF diversity in field VMD [14].

We detected interesting differences between the years. In the first years, mineral fertilizer was used and only from the beginning of 2010 pig manure was used as a fertilizer. This might explain differences in fungal communities observed between 2008 and 2010. Previously, it has been shown that different types of fertilizer treatments contribute to different microbial communities [45]. Notably, in our study we detected more ascomycetes and less basidiomycetes and fungi in general in 2010 compared to 2008 in both fields (Fig. 1) which might be an indication of changed community structure due to changed fertilizer treatment. Also the diversity and richness of AMF was higher in 2010.

Community structure and diversity of soil fungi are important determinants of key soil ecosystems functions such as decomposition of organic matter. Indeed, we could detect a correlation between community structure of fungi and decomposition-related enzyme activities. Moreover, the combination of phylogenetic analyses with functional assays proved highly useful, providing a more complete picture of fungal community dynamics. We found a correlation between Mn-peroxidases produced and the ascomycete diversity (and richness). Mn-peroxidases can be produced primarily by basidiomycetes as well as some ascomycetal groups [46]. However, not much is known of the ecology. AM fungi are strongly affected by agricultural practices and changes in soil characteristics [47]–[49] such as moisture and manure addition. Indeed, we saw an increase of AMF diversity in 2010 when the fertilizer was changed from mineral to pig manure which is in correspondence with results from Verbruggen *et al*. [14] who found organic fertilizers having a positive effect on AMF diversity.

Only few studies have evaluated the potential impacts of GM-plants in the context of impacts of multiple cultivars on fungal rhizosphere communities. Most of them have found some degree of cultivar dependence of soil fungal community composition [13], [18], [47] while another one [20] found no cultivar dependent alterations in the fungal communities. We found some indications of cultivar dependence, for instance the cultivar ‘Premiere’ had a lower amount of fungi, as measured by ergosterol, in its rhizosphere than two other cultivars ‘Aveka’ and ‘Désirée’ Despite some differences in enzymatic activities, total fungal diversity was not affected by the cultivar-type at the level of OTUs and orders. Ascomycetal diversity was affected at the level of classes as one cultivar, ‘Désirée’, had a less diverse community in its rhizosphere. To conclude, we found some degree of cultivar dependence in measured parameters at some time points, but these differences were mostly not persisting over time and not observed in both fields, similarly as found by Weinert et al. [18].

In this study the GM-variety ‘Modena’ was not significantly different from its parental variety ‘Karnico’ in any measured parameter and it seemed that these cultivars had a very similar effect on both the structure and function of soil fungal communities. The only significant effect was the difference in the amount of fungi in the rhizosphere of the two cultivars in the field VMD during senescence, in all years of the study. This was, however, seen only in one of the two soils studied and can, thus, be ruled out as a cultivar-soil interaction effect. There was no overall trend of multiple parameters being consistently changed by any of the cultivars while the other factors (i.e. growth stage and season) had consistent effect on multiple parameters measured.

The growth stage can also affect the outcome of the comparison between the cultivars. Other authors have found differences in microbial communities associated with GM-potatoes mostly at the senescent growth stage [19], [33], [40], [50], [51]. The soil micro-organisms have an important role in soil ecosystem functioning such as decomposition of plant residues and nutrient cycling [52]. Thus it is possible that the differences at the stage of senescence as found in this study could lead to changes in function and might, thus, have long lasting effects. In this study, all analyses indicated that when the fungal communities were assessed after removal of the plant or in the rhizosphere of the next crop in rotation, there were no differences between fungal communities from field plots that contained harvested modified potato plants. So, we did not detect any significant connection between the previous cultivar of potato on the fungi in the rhizosphere of the next crop barley. Hence, the changes in the fungal biomass associated with starch modified potato plants detected at certain time points and fields in this study were temporary and did not persist into the next field season. A similar observation was made for bacteria after cropping of transgenic canola [53].

In conclusion, plant growth stage, year and field site were the factors contributing most to variation in the potato-associated fungal communities. Despite some differences in fungal-related parameters between individual cultivars, there were no directional effects and most of the differences observed were not consistent between fields and years. Even at the level of individual OTUs, there were no consistent significant differences between cultivars in community structure and no differences in community function were found during and after the growth of the plant. However, as was seen from conflicting evidence between different studies, we acknowledge that potential effects of GM-crops on soil fungal communities vary between crop species and types of modifications done to the plant making a case-by–case evaluation strategy advisable. We hypothesized that this modification would have no direct but rather indirect unintended effects of the modification on the plant physiology through production of different exudates. Data presented in this study allowed us to conclude that the modification studied here has no long-lasting effects on soil fungal communities and that the potato plant growth stage, season and field location affect the soil fungal community structure and function more than the cultivar-type or starch modification of tubers.

## Supporting Information

Figure S1 **Principal component analysis of function and diversity of fungal communities in between growth stages, fields and years.** Field BUI is marked with closed symbols and solid lines while field VMD with open symbols and dotted lines. Year 2008 is marked with black markers, year 2009 with red markers and 2010 with blue marker. The explanatory parameters are mentioned next to the axis.
(TIF)

Table S1 **Soil characteristics and fertilizers added to the fields**. In the fertilizer treatments CAN  = Calcium Ammonium Nitrate, NP = nitrogen as ammonium sulphate and phosphorous as P_2_O_5_ and ORG = organic fertilizer = pig manure.
(XLS)
