# Supplementary figures and images for: A 3-Year Study Reveals That Plant Growth Stage, Season and Field Site Affect Soil Fungal Communities while Cultivar and GM-Trait Have Minor Effects
Source: PLoS One. 2012 Apr 17;7(4):e33819. doi: 10.1371/journal.pone.0033819 (PMC3328480; doi:10.1371/journal.pone.0033819)

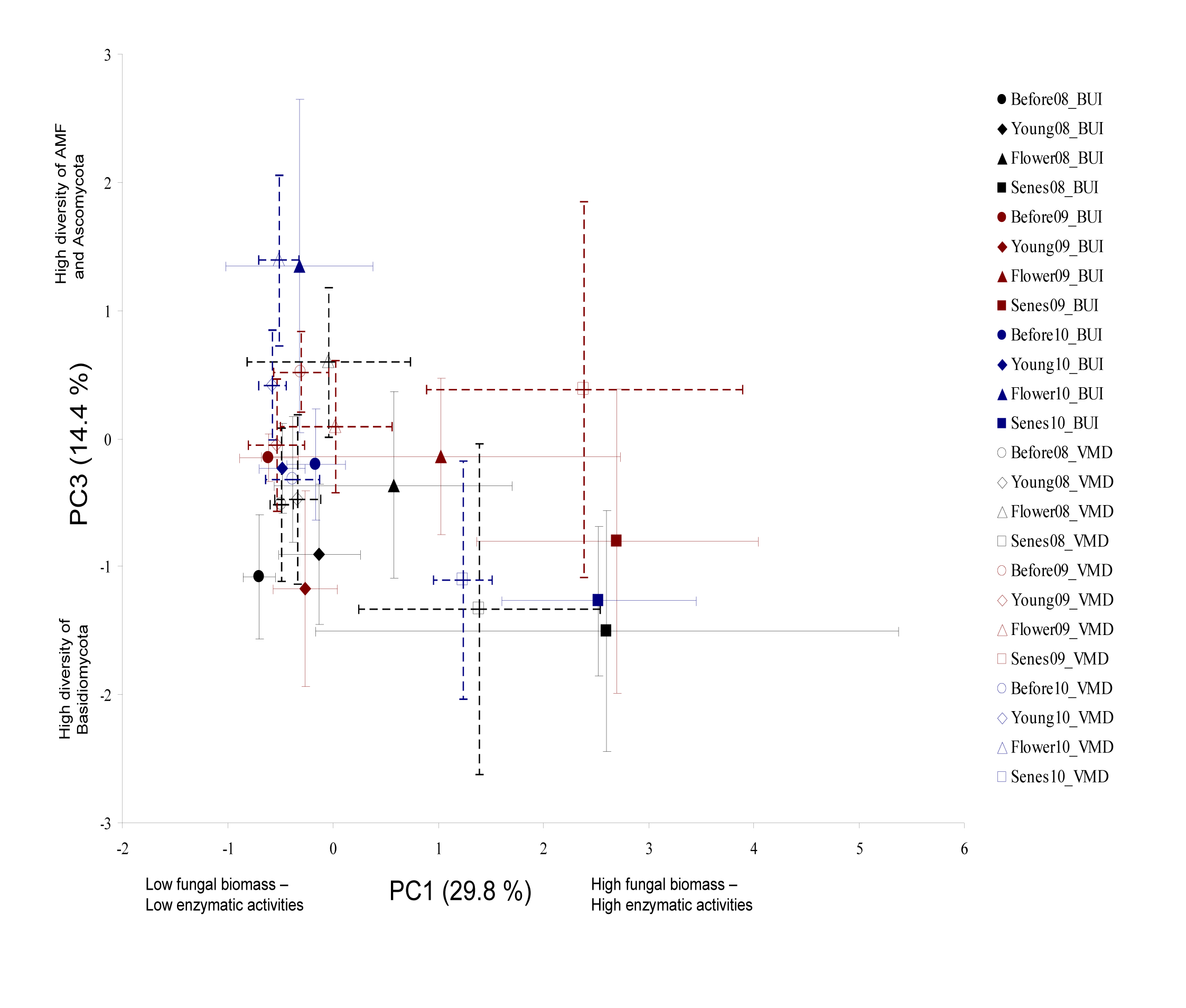

Supplement: Figure S1 — Principal component analysis of function and diversity of fungal communities in between growth stages, fields and years. Field BUI is marked with closed symbols and solid lines while field VMD with open symbols and dotted lines. Year 2008 is marked with black markers, year 2009 with red markers and 2010 with blue marker. The explanatory parameters are mentioned next to the axis. (TIF) [file pone.0033819.s001.tif]
